# Effect of Perioperative Prophylactic Intravenous Antibiotic Use in Immediate Implant-Based Breast Reconstruction: A Retrospective Matched Cohort Study

**DOI:** 10.1055/a-2161-7521

**Published:** 2024-02-07

**Authors:** Seok Kyung In, Seok Won Park, Yujin Myung

**Affiliations:** 1 Seoul National University Bundang Hospital, Seoul National University College of Medicine, Seongnam-si, Republic of Korea

**Keywords:** breast implants, infections, antibacterial agents

## Abstract

**Background** Among breast reconstruction methods, implant-based breast reconstruction has become the mainstream. However, periprosthetic infection is still an unresolved problem. Although published articles have revealed that limited use of antibiotics is sufficient to reduce infection rates, the number of surgeons still preferring elongated usage of antibiotics is not less. The aim of our study is to validate the appropriate duration of antibiotic use to reduce infection rate after implant-based breast reconstruction.

**Methods** A retrospective study reviewed medical record of 235 patients (274 implants for reconstruction) who underwent prepectoral direct to implant breast reconstruction using acellular dermal matrix wrapping technique. Infection rates were analyzed for the patients administered postoperative prophylactic antibiotics until drain removal and those who received only perioperative prophylactic antibiotics for 24 hours.

**Results** Of the 274 implants, 98 who were administered prophylactic antibiotics until drain removal had an infection rate of 3.06% (three implants) and 176 who received prophylactic antibiotics no longer than 24 hours postoperatively had an infection rate of 4.49% (eight implants). A total of 11 patients diagnosed with postoperative infection clinically, 8 were salvaged by antibiotic treatment, and 3 had implant removal and replacement with autologous flap. Postoperative antibiotic prophylaxis duration had no statistically significant effects in the risk of infection ( *p* = 0.549).

**Conclusion** The duration of prophylactic antibiotics after surgery was not related to infection risk. Further study with a large number of patients, randomized control study, and route of antibiotics is needed.

## Introduction

Implant-based breast reconstruction is the most common method of breast reconstruction. 1 According to U.S. statistics, 137,808 breast reconstructions were performed in 2020. Of these, 105,665 were immediate reconstructions and 96,300 used silicone implants. 2 Immediate breast reconstructions using implants were performed in 70% of cases. In Korea, comparable results have been recently published, with 4,702 of 7,088 breast reconstructions (66%) performed using implants. 3 Over the past few years, subpectoral implant placement has been generally replaced by prepectoral reconstruction. 4 In an effort to reduce the capsular contracture rate, the acellular dermal matrix (ADM) wrapping technique has been recently developed. 5

In the case of implant-based breast reconstruction, the advantages of simplicity and low patient burden are always accompanied by the risk of prosthetic-related infection. In severe cases, surgical site infection can lead to explantation and a worst-case outcome for the patient. 6 Infections range from self-limiting to implant failure and sepsis. 7 8 Subclinical infection from endogenous flora within the nipple mammary duct is currently accepted as one of the causes for capsular contracture. 9

Published infection rates after implant-based reconstruction range from 0 to 29%, with an average of approximately 5.83%. 10 11 12 Because the complication rate is higher in implant-based than in autologous tissue-based reconstruction, conversion to autologous tissue may later become necessary. 12 13 Therefore, many studies have focused on reducing infection rates. Surgical factors including antibiotic breast irrigation, no-touch technique, and nipple shields have been reported as effective measures. 14 15 Among the perioperative elements, prophylactic antibiotics have reduced postoperative infection and implant failure rates, but there is conflicting evidence regarding the dosage and duration. 16 17 One study reported that the infection rate increased when preoperative antibiotics alone were administered. 18 Another found that for breast reconstruction, half of the doctors used prophylactic antibiotics only preoperatively, and the other half administered them until drain removal. 19

For clean and clean-contaminated procedures, the Centers for Disease Control and Prevention (CDC) guidelines recommend that prophylactic antibiotics should not be administered after the surgical incision is closed, even in the presence of a drain. 20 These guidelines are not specific to breast surgery; however, one study has classified breast surgery as a clean-contaminated surgery, because the mammary duct is damaged and normal flora is present at the site. 21

Prophylactic antibiotic regimen changes were evaluated by the Korean Health Insurance Review and Assessment Service (HIRA). The aim was to prevent infection at the surgical site and avoid antibiotic misuse and abuse by encouraging appropriate agent selection and treatment period. Implant-based breast reconstruction, the subject of this study, was included under the breast surgery classification. HIRA's recommendations were (1) prophylactic antibiotics should be administered, (2) the first prophylactic antibiotic dose should be given within 1 hour prior to the skin incision, and (3) prophylactic antibiotic administration should be discontinued within 24 hours of surgery. According to these recommendations, our institution began a study to evaluate the appropriateness of prophylactic antibiotic use in surgery. For breast reconstruction, as of October 2020, the surgical protocol was maintained; however, the discontinuation of prophylactic antibiotic administration was changed from the time of drain removal to the day of operation. Cases for the study accumulated over time, and the requirements for a matched cohort study were well met; thus, this study was conducted.

## Methods

This was a retrospective cohort study of all consecutive implant-based breast reconstructions using ADM after mastectomy at our institution between July 2019 and December 2021.

**Fig. 1:**
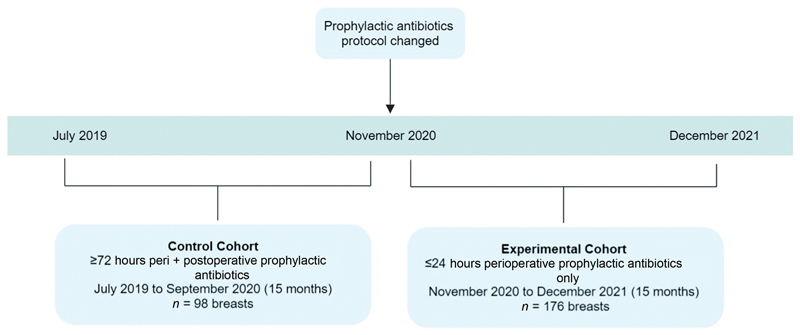
Control and experimental cohort timeline.

During the 3-year period, 235 women ( *n* = 274 breasts) underwent direct implant breast reconstruction using the ADM wrapping technique. Two cohorts were selected for reconstruction using silicone implants. The patients in the first cohort (Cohort 1) were administered postoperative prophylactic antibiotics until drain removal. Patients in the second cohort (Cohort 2) received only perioperative antibiotics (up to 24 h from the procedure; Fig. 1 ).

### Prophylactic Antibiotic Patient Cohorts

All patients were preoperatively administered first-generation cephalosporins intravenously. Cohort 1 patients continued to receive antibiotics postoperatively until suction drain removal. These were given intravenously throughout hospitalization (Cefazolin 1 g every 8 h) and peroral (Cefadroxil 500 mg every 8 h) after discharge and until drain removal (average 9.6 ± 2.4 d).

Cohort 2 patients received antibiotics (Cefazolin) only for a maximum of 24 hours following surgery.

### Surgical Techniques

Surgical disinfection techniques were performed according to an identical protocol. Immediately after mastectomy, all surgical fields were disinfected with povidone-iodine solution, and breast pockets were irrigated thoroughly with povidone-iodine solution and normal saline. A sheet of 16 × 16 cm ^2^ or 18 × 18 cm ^2^ ADM was used to wrap the selected implants. The ADM-wrapped implants were inserted into the breast pocket within the prepectoral layer using the no-touch technique. Two closed suction drains were placed in all the patients. One drain was placed inferior to the implant and the second was placed lateral to the implant, in the axillary direction. Drains were removed when each drainage was less than 25 mL per day for two consecutive days.

### Management of Postoperative Infection

**Fig. 2:**
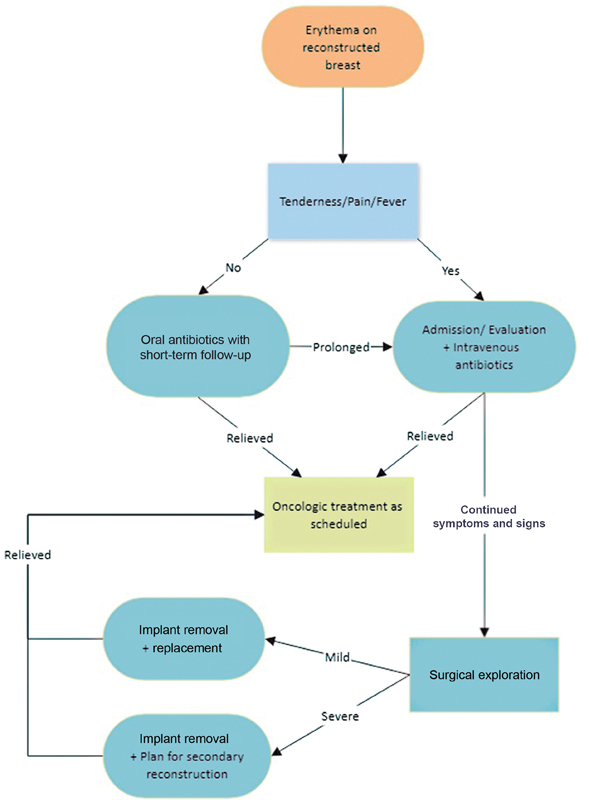
Breast implant infection management protocol.

During follow-up, infections were noted with a clinical diagnosis. The following signs and symptoms were presumed to stem from infection: erythema, drainage with purulence, febrile condition with clinical manifestations, and localized pain and tenderness. Localized erythema of the breast could be “red breast syndrome,” and difficult to differentiate from infection; thus, antibiotic treatment (cefazolin, gentamycin, vancomycin, clindamycin) was given. If flap necrosis or prolonged discharge did not resolve with antibiotic treatment alone, the implant was removed. Except for patients not desiring reconstruction, most patients converted to autologous flaps (latissimus dorsi rotation flap and free transverse rectus abdominis flap) after implant removal. The postoperative breast implant infection management protocol is shown in Fig. 2 .

### Statistical Analysis

For each factor, the Fisher's exact test, Student's *t* -test, chi-square test, or logistic regression test was performed as appropriate. Statistical analysis was conducted using SPSS version 22.0 (SPSS Inc., Chicago, IL). In all statistical comparisons, a *p* -value of <0.05 was considered to indicate statistical significance.

## Results

Over the 3-year period, 235 patients underwent 39 bilateral and 196 unilateral direct to implant breast reconstructions with the ADM wrapping technique, totaling 274 breast reconstructions for both the cohorts. Eleven patients required readmission, prolonged antibiotic treatment, or surgical intervention (4.01% of all enrolled patients). In Cohort 1, who received postoperative antibiotics, there were three infections; in Cohort 2, receiving perioperative prophylaxis only, there were eight infections (3.06 and 4.49%; *p* = 0.549).

Among the infection cases, there were three that did not resolve with antibiotic treatment, so that it was necessary to return to the operating room. Studies have offered various definitions of infection, but those requiring reoperation are considered major infections. 8 Thus, the major infection rate in our study was 1.02% (one case) in Cohort 1 (receiving peri- and postoperative prophylaxis) and 1.18% (two cases) in Cohort 2 (perioperative prophylaxis ≤24 h), similar to the overall results.

The two cohorts varied only according to the timeframe of the procedure (before or after November 2020) and the antibiotic regimen; they did not differ significantly in age, body mass index (BMI), or preoperative chemotherapy, or radiotherapy. In terms of treatment, the number of patients receiving postoperative radiation therapy increased over time, and suction drains tended to be removed later. The mastectomy resection amount was almost the same between groups, but there was a tendency to insert smaller implants in Cohort 2. Other postoperative complications such as seroma, hematoma, wound dehiscence, and partial necrosis occurred insignificantly. Furthermore all the patients with these complications actually did not progress to infection clinically ( Table 1 ).

**Table 1:** Baseline patient characteristics

Characteristics	Total patients	≥72 hour peri + post-op ABX	≤24 hour peri-op ABX only	Statistical comparison ( *p* )
Infection rate ( *n* = 274)	11/274	3/98 (3.06%)	8/176 (4.49%)	0.549
Age, years	46.2 ± 7.9	46.1 ± 7.9	46.3 ± 7.9	0.899
BMI, kg/m ^2^	21.9 ± 3.0	21.8 ± 2.7	21.9 ± 3.1	0.753
Hypertension	3	0/98 (0%)	3/176 (1.70%)	0.263
Diabetes	1	0/98 (0%)	1/176 (0.57%)	0.642
Preoperative irradiation	8/274	4/98 (4.08%)	4/176 (2.27%)	0.394
Preoperative chemotherapy	33/274	12/98 (12.24%)	21/176 (11.93%)	0.939
Postoperative irradiation	90/274	24/98 (24.49%)	66/176 (37.5%)	** 0.028 a**
Postoperative chemotherapy	73/274	26/98 (26.53%)	47/176 (26.70%)	0.975
Excised breast tissue weight, g	196.7 ± 107.4	196.4 ± 104.3	196.8 ± 106.2	0.973
Implant size, mL	238.1 ± 85.2	258.2 ± 88.2	226.9 ± 81.5	** 0.003 a**
Removal of last H-vac drain, POD	11.2 ± 3.0	9.6 ± 2.4	12.9 ± 11.5	** 0.005 a**
Op time, minutes	231.3 ± 74.7	219.3 ± 72.2	237 ± 75.5	** 0.047 a**
Seroma	4/274	1/98 (1.02%)	3/176 (1.70%)	0.549
Hematoma	2/274	0/98 (0.00%)	2/176 (1.14%)	0.873
Dehiscence	8/274	1/98 (1.02%)	7/176 (3.98%)	0.154
Partial necrosis	12/274	6/98 (6.12%)	6/176 (3.41%)	0.910

Abbreviations: ABX, antibiotics; BMI, body mass index; Op, operative; peri-op, perioperative; POD, postoperative day; post-op, postoperative.

Note: Bold *p* -values reflect statistical significance. Statistical comparison according to risk factors.

a Statistically significant.

### Noninfection versus Infection Patients

The proportion of infected to total patients (11/263) was low and thus, it was difficult to obtain statistically significant results on comparative analysis ( Fig. 1 ). Some notable factors were BMI, excised breast tissue weight, and implant size. In the infection group, average BMI was higher, the weight of excised breast tissue was approximately 54.8 g heavier, and the implant size was 36.6 mL larger. However, these differences were not statistically significant ( Table 2 ). On the other hand, logistic regression test shows that age, BMI, operation time, excised breast tissue weight, and implant size, and the day before the removal of last Hemovac (H-vac) drain are not statistically significant ( Table 3 ).

**Table 2:** Statistical analysis between noninfected and infected groups

Characteristics	Noninfected patients ( *n* = 263)	Infected patients ( *n* = 11)	Statistical comparison ( *p* )
Age, years	46.2 ± 7.9	46.3 ± 9.3	0.973
BMI, kg/m ^2^	21.8 ± 3.0	23.3 ± 3.0	0.106
Prophylactic ABX			0.549 (OR)
≥72 hours peri + postoperative ABX	168	8	
≤24 hours perioperative ABX only	95	3	
Preoperative irradiation	7/263 (2.66%)	1/11 (9.09%)	0.963
Preoperative chemotherapy	32/263 (12.17%)	0/11 (0%)	0.248
Postoperative irradiation	89/263 (33.84%)	1/11 (9.09%)	0.075
Postoperative chemotherapy	83/263 (31.56%)	2/11 (18.18%)	0.282
Excised breast tissue weight, g	194.5 ± 105.0	249.3 ± 106.9	0.091
Implant size, mL	236.6 ± 84.2	273.2 ± 104.5	0.163
Removal of last H-vac drain, POD	11.7 ± 9.6	11.5 ± 4.3	0.952
Op time, minutes	232.4 ± 75.3	203 ± 55.1	0.211

Abbreviations: ABX, antibiotics; BMI, body mass index; Op, operative; OR, odds ratio; periop, perioperative; POD, postoperative day; postop, postoperative.

**Table 3:** Statistical analysis of infection and risk factors with logistic regression test

Risk factor	*p* -Value
Age, years	0.749537
BMI, kg/m ^2^	0.385135
Op time, minutes	0.083956
Excised breast tissue weight, g	0.554289
Implant size, mL	0.321674
Removal of last H-vac drain, POD	0.313679

Abbreviations: BMI, body mass index; Op, operative; POD, postoperative day.

### Microbiology and Antibiotics

In most infection cases, the implant was salvaged through antibiotic treatment alone. The bacterial strains cultured from infections included methicillin-susceptible coagulase-negative staphylococci (two cases), methicillin-susceptible *Staphylococcus aureus* (one case), and methicillin-resistant *Staphylococcus aureus* (one case). We administered vancomycin (1 g intravenously every 12 h) as an empirical antibiotic to patients with suspected infections.

## Discussion

In our study, the duration of prophylactic antibiotics did not correlate with the postoperative infection rate after immediate breast reconstruction ( *p* = 0.549). Therefore, our results corroborate the CDC guidelines for surgical site infection and the HIRA protocol. 20 They also corroborate the findings of Phillips et al, who also reported no benefit in patients who received >24 hours of postoperative antibiotics. 10

In our study, the total infection rate was 4.01% over the 3-year study and the mean 1.5-year follow-up periods. In our study population, there were only three infection cases (1.09%) that caused concerns regarding a need for salvage due to implant exposure or uncontrolled infection. Most localized erythema was resolved with empirical antibiotic treatment. Our infection rate was lower than previously reported rates; this is probably due to the lower BMI and smaller implant size compared with those in Caucasians. 16 Because our study is the most recent, we assume that there has been a corresponding decrease of infection rates in other institutions, due to improved patient management and surgical techniques.

In our study, most of the infections manifested as breast erythema. When localized redness is present, skin flap cellulitis needs to be distinguished from a hyperreaction to ADM, i.e., red breast syndrome. 22 Vancomycin was considered an appropriate choice for empirical treatment because the highest bacterial strain ratio belongs to gram-positive pathogens, including staphylococci. 23 24 However, infections with gram-negative pathogens such as *Acinetobacter baumannii* , *Pseudomonas aeruginosa* , and even *Mycobacterium* , have been reported. Thus, clinical judgement may suggest addition of another appropriate antibiotic agent. 6

Although not statistically significant in our study, the factors that correlated with infection were increased BMI, excised breast tissue weight, and implant size. The relation of BMI with infection or complications is well-known from previous studies. Antony et al, have described higher BMI as a significant independent risk factor for developing complications during breast reconstruction. 23 24 25

The longer the follow-up period, the more the infection may occur after implant reconstruction. There are limitations for difference in follow-up period between the two groups and the number of patients. However all the infection cases in our study occurred within postoperative 1 year even in Cohort 1. Capsular contracture is another accepted cause of subclinical infection. However, because this takes a considerable period after surgery to develop, a long-term follow-up study is needed. Samanta et al reported that after 10 years of observation, close to 44.9% of cases required implant removal due to reconstruction failure. 6 27 Therefore, studies focused on immediate or mildly delayed infection rates, like this one, are insufficient to determine whether prophylactic antibiotics are suitable. Given the lower infection rates in this study, a much larger cohort population is required to determine the optimal duration for prophylactic antibiotics. In addition, this study was not a prospective, double-blind, controlled study, and only evaluated two cohorts. Nevertheless, our study offers interesting results, because of the cohorts that matched in all variables except the time period of surgery and the antibiotic administration regimen.
